# DISPARITIES IN MOCA AND MMSE SCORES AMONG DIVERSE OLDER ADULTS: A CASE ANALYSIS OF CREOLE-SPEAKING PARTICIPANTS

**DOI:** 10.1093/geroni/igad104.2601

**Published:** 2023-12-21

**Authors:** Marie Adonis-Rizzo, Ruth Tappen, Monica Rosselli, David Newman, Somi Panday, Joshua Conniff

**Affiliations:** Florida Atlantic University, Lake Worth, Florida, United States; Florida Atlantic University, Boca Raton, Florida, United States; Florida Atlantic University (FAU), Boca Raton, Florida, United States; Florida Atlantic University, Boca Raton, Florida, United States; Florida Atlantic, Boca Raton, Florida, United States; Florida Atlantic University, Boca Raton, Florida, United States

## Abstract

Almost 11% of older adults in the US have Alzheimer’s or related dementia. Most studies suggest that those who are Black or Hispanic are at higher risk. A culturally diverse sample, including Haitian older adults, was recruited from South Florida communities to participate in a longitudinal study, “In-Vehicle Sensors to Detect Change in Cognition of Older Drivers.” An extensive cognitive battery was administered for comparison with driving behaviors. No validated translations in Creole were available, so three Haitian-born examiners translated and back-translated test items into Creole. Two additional bilingual examiners verified the results. An analysis of variance (ANCOVA) comparing Haitian participants (N=12) and European Americans (N=135) on the MoCA controlling for age and education indicated the European American group scored higher (M = 25.78, SD = 2.57) than the Haitian Group (M =24.17, SD = 3.01), a significant difference in test performance, F (1, 124) = 6.11, P= .015. Results on four subscales of the MoCA evidenced significantly higher scores for the European American group: Visual EX Total, F (1, 124) = 22.52, p < .001; Serial-7, F (1, 124) = 12.09, p <.001; Attention Total, F (1, 124) = 5.10, p <.001; Letter Score F (1, 124) = 13.09, p <.001. No differences in language, abstract, delayed recall, or orientation were found. These preliminary results suggest cultural influences may account for differences in test performance. Further investigation with a larger sample of Haitian older adults is needed to explain differences in performance on this widely used test.

